# Development of a gene synthesis platform for the efficient large scale production of small genes encoding animal toxins

**DOI:** 10.1186/s12896-016-0316-3

**Published:** 2016-12-01

**Authors:** Ana Filipa Sequeira, Joana L. A. Brás, Catarina I. P. D. Guerreiro, Renaud Vincentelli, Carlos M. G. A. Fontes

**Affiliations:** 1 Centro Interdisciplinar de Investigação em Sanidade Animal (CIISA) - Faculdade de Medicina Veterinária, Universidade de Lisboa, Avenida da Universidade Técnica, Lisboa, 1300-477 Portugal; 2 NZYTech Genes & Enzymes, Campus do Lumiar, Estrada do Paço do Lumiar, Edifício E, r/c, Lisboa, 1649-038 Portugal; 3 Unité Mixte de Recherche (UMR) 7257, Centre National de la Recherche Scientifique (CNRS) – Aix-Marseille Université, Architecture et Fonction des Macromolécules Biologiques (AFMB), Campus de Luminy, 163 Avenue de Luminy, Marseille, CEDEX 09 13288 France

**Keywords:** Gene synthesis, Assembly PCR, Gene design, Venom peptides

## Abstract

**Background:**

Gene synthesis is becoming an important tool in many fields of recombinant DNA technology, including recombinant protein production. *De novo* gene synthesis is quickly replacing the classical cloning and mutagenesis procedures and allows generating nucleic acids for which no template is available. In addition, when coupled with efficient gene design algorithms that optimize codon usage, it leads to high levels of recombinant protein expression.

**Results:**

Here, we describe the development of an optimized gene synthesis platform that was applied to the large scale production of small genes encoding venom peptides. This improved gene synthesis method uses a PCR-based protocol to assemble synthetic DNA from pools of overlapping oligonucleotides and was developed to synthesise multiples genes simultaneously. This technology incorporates an accurate, automated and cost effective ligation independent cloning step to directly integrate the synthetic genes into an effective *Escherichia coli* expression vector. The robustness of this technology to generate large libraries of dozens to thousands of synthetic nucleic acids was demonstrated through the parallel and simultaneous synthesis of 96 genes encoding animal toxins.

**Conclusions:**

An automated platform was developed for the large-scale synthesis of small genes encoding eukaryotic toxins. Large scale recombinant expression of synthetic genes encoding eukaryotic toxins will allow exploring the extraordinary potency and pharmacological diversity of animal venoms, an increasingly valuable but unexplored source of lead molecules for drug discovery.

**Electronic supplementary material:**

The online version of this article (doi:10.1186/s12896-016-0316-3) contains supplementary material, which is available to authorized users.

## Background

Synthetic biology, an interdisciplinary branch of biology, is quickly becoming one of the most attractive areas of research thanks to the recent developments in gene synthesis technology. In combination with intelligent gene design, gene synthesis is emerging as a valuable tool to support recombinant protein expression. *De novo* gene design allows optimizing codon usage to the recombinant host system thus promoting the effective operation of the cellular translational machinery. In addition, in cases where the nucleic acid template is not available, gene synthesis allows creating DNA molecules *de novo*. The exponential growth of genomic and metagenomic databases and the current limitations in using this highly useful sequence information due to the lack of tangible DNA are promoting the rapid development of novel gene synthesis technologies.

In recent years, a variety of gene synthesis methodologies have been developed based on the assembling of oligonucleotides into complete genes. Early approaches advanced to synthesize nucleic acids used the enzymatic ligation of pre-formed duplexes of phosphorylated overlapping oligonucleotides [1]. Subsequently, self-priming PCR [2], PCR assembly [3], Polymerase chain assembly (PCA) [4] and template-directed ligation [5] were described as efficient concepts for *de novo* gene synthesis of nucleic acids. Recently, methods based on a two-step approach were reported for the production of long DNA sequences. Examples of these technologies are the PCR-based thermodynamically balanced inside-out technology (TBIO) [6], the two-step total gene synthesis method [7] that combines both dual asymmetrical PCR (DA-PCR) and overlap-extension (OE-PCR), the PCR-based two-step DNA synthesis (PTDS) [8] and PCR-based accurate synthesis (PAS) [9].

Lately, improvements in PCR-based gene synthesis methods, as exemplified by the development of the improved PCR synthesis (IPS) and the simplified gene synthesis (SGS) protocols [8, 9], have been described and incorporate significant simplifications over earlier strategies. SGS uses oligonucleotides of 40 nucleotides (nt) in length and 18–20 nt of overlap region, which are assembled in a unique PCR-assembly reaction leading to the direct construction of the full-length DNA molecule. The simplicity of this protocol combined with its relative low cost, since there no requirement for phosphorylation or purification of the oligonucleotides exists, are a solid base for the development of even more effective PCR-based methods. However, major drawbacks persist and effective improvements need to be implemented in current synthetic protocols to allow their translation to a large scale. One of the major bottlenecks of current gene synthesis protocols consists on the quality of the oligonucleotides used for nucleic acid assembly. It is known that all current gene synthesis methods accumulate errors in the final synthetic molecules. Sequence errors usually derive from the incorporation of imperfect synthetic oligonucleotides or result from low fidelity rates associated with the enzymatic assembling step. Current oligonucleotide synthesis methods produce sequences that are often prematurely terminated, or comprise internal mutations (error rates range from 1 to 10 mutation per kilobase (kb)) [10]. In addition, chemical synthesis of DNA molecules usually not only involve moderate to high error rates but also high costs. Moreover, the chemical synthesis of a desired gene also depends on the accuracy of the DNA polymerase used to assemble the oligonucleotides in a final DNA sequence. Therefore, DNA errors are inevitable and it is often necessary to remove the incorrect synthetic DNA molecules using enzymatic methods [11, 12]. Improvements in oligonucleotide quality, error correction and DNA polymerase efficacy are thus urgently required.

Conventionally, PCR-based gene synthesis is employed to produce a single gene at a time. Thus, development of automated platforms that effectively generate large libraries of nucleic acids is urgently needed. The different steps leading to a single PCR-assembly strategy need to remain simple, accurate and robust when extended to the assembly of multiple genes simultaneously. To develop large scale methods, many factors that affect the efficiency of gene assembly, such as DNA polymerases performance or oligonucleotide concentration and quality require optimization. This work describes different approaches carried out to optimize current gene synthesis protocols. The data was integrated to develop a novel platform which was applied to efficiently synthesize and clone a large number of nucleic acids encoding venom peptides. This automated platform can be translated to the rapid generation of complex gene libraries encoding different families of biotechnologically relevant and valuable proteins and peptides.

## Methods

### Gene design

The original purpose of this research was to optimize protocols for the synthesis of small genes encoding eukaryotic peptides for expression in *Escherichia coli*. Three genes of different lengths (A: 290 bp, B: 260 bp, and C: 329 bp) were selected to develop these studies. Gene A encodes the alpha-elapitoxin-Nk2a toxic protein isolated from the *Naja kaouthia* venom, and genes B and C encode two different venom peptides of unknown function. Venom genes were designed by back-translating the peptide sequence and optimizing codon usage for high levels of expression in *E. coli*. Guanine-cytosine (GC) content was set to vary between 40 and 60%. Gene design maximized stable mRNA molecules, minimized the presence of repeated sequences and avoided the appearance of *E. coli* regulatory sequences such as promoters, activators or operators. In addition, Codon Adaptation Index (CAI) value was set to be higher than 0.8. DNA sequences of the three genes are presented in Additional file 1: Table S1.

### Oligonucleotides and purification

Oligonucleotides were synthesized by three different suppliers (A, B and C) using the smaller scale available with standard desalting. Reverse-phase cartridge and reverse-phase HPLC purifications were also tested for oligonucleotides obtained from supplier C. Each oligonucleotide was reconstituted in 10 mM Tris-HCl (pH 8.5) to a 100 μM final concentration and kept at −20 °C. Oligonucleotides were used individually or in mixtures at concentrations described below.

### Primer design

**Fig. 1:**
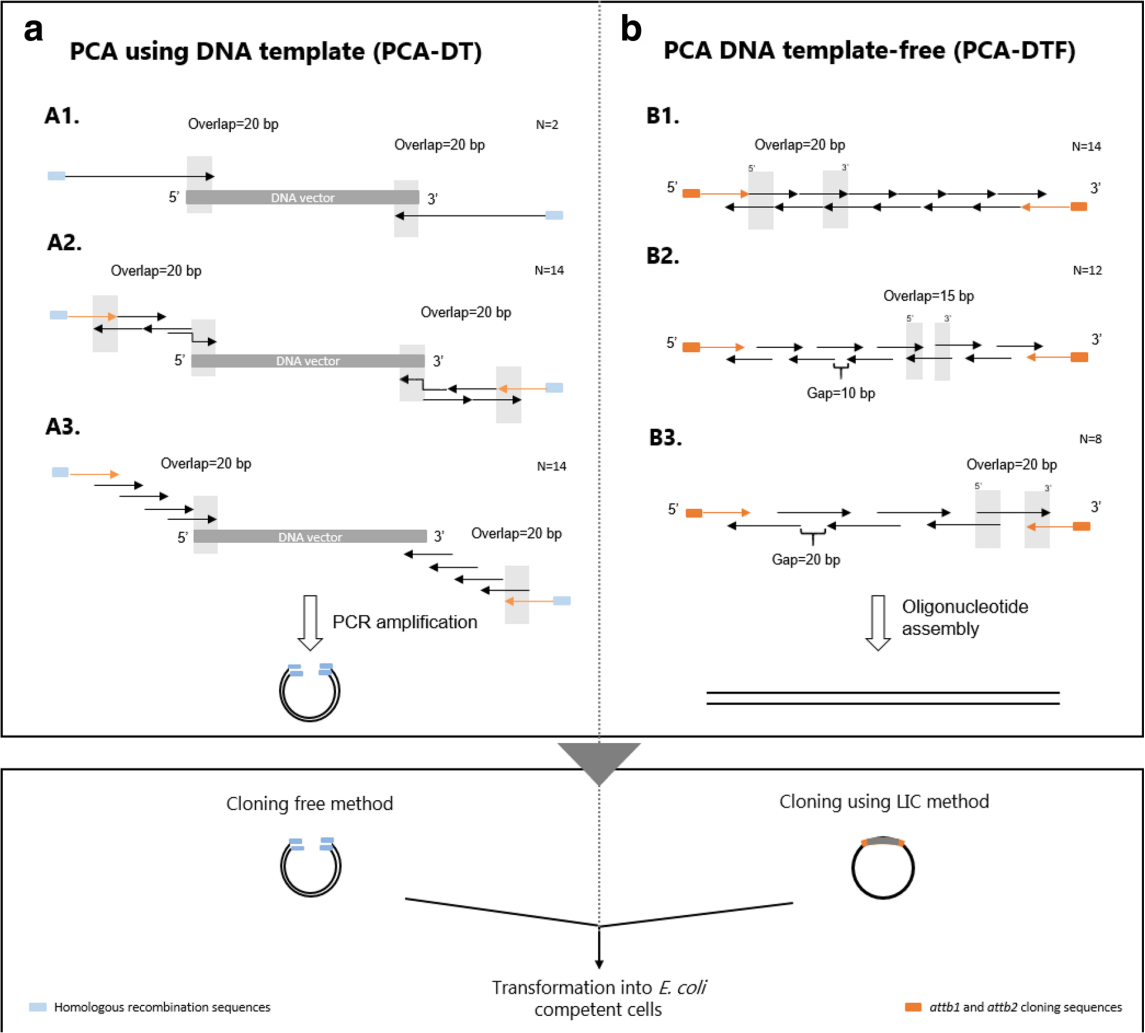
Schematic diagram representing the two different approaches used for gene synthesis: **a**. Polymerase chain assembly using DNA template (PCA-DT), and **b**. Polymerase chain assembly DNA template-free (PCA-DTF). Each oligonucleotide is represented as an arrow; black arrows correspond to internal (inner) oligonucleotides while external (outer) oligonucleotides are denoted as orange arrows. Each strand of the desired gene is dissected into two or more oligonucleotides that are amplified (A) or overlap extended (**b**) by a DNA polymerase. A1-Two long oligonucleotides were designed with a 20 bp overlap region with the cloning vector; A2-Two sets of 40-mer oligonucleotides containing the 5’-end and 3’-end sequences of the desired gene were assembled by PCR; A3 – Successive oligonucleotides with 40 nt in length were designed with a 20 nt overlaps regions between adjacent oligonucleotides to construct the full-length of cloning vector containing the desired gene. The PCR products from A1, A2 and A3 strategies were inserted in *E. coli* cells through homologous recombination. Strategy B corresponds to a single-step PCR assembly step that does not require template DNA. B1 – Gapless 40-mer oligonucleotides containing 20 nt overlap regions between primers were used to assemble the synthetic gene; B2 – 40-mer oligonucleotides with 15 bp overlaps between forward and reverse primers, with gaps of 10 nt were mixed to produce the gene of interest; B3 – The synthetic gene was synthesized by mixing a 60-mer overlapping oligonucleotides containing gaps of 20 nt. After overlap extension by DNA polymerase, gene fragments from B1, B2 and B3 were cloned into a vector using a ligation-independent cloning method. All outer primers contain a vector complementary region at the 5’-end (highlighted in orange rectangles) or a 16 bp complementary sequence (highlighted in blue rectangles) to facilitate the cloning reaction

The DNA sequence of each gene was used as a template to design the assembly oligonucleotides by dividing the entire sequence into overlapping primers with defined lengths. The external oligonucleotides, termed outer primers, correspond to the external forward and reverse primers. For strategy A (see below), the outer primers include a complementary sequence of 15 bp to promote plasmid re-circulation through homologous recombination (Fig. 1a). In strategy B (see below) the outer primers contained an additional sequence at the 5’-terminus of both forward and reverse primers for cloning into the cloning vector through a Ligation-Independent Cloning protocol (LIC) (Fig. 1b). Internal oligonucleotides (termed inner primers) are used in higher numbers than outer primers. Depending on the gene assembly strategy used, the oligonucleotides were designed with gaps between adjacent primers and comprising 15–20 bp overlap regions. The sequence of all oligonucleotides used in these studies are displayed in Additional file 2: Table S2.

### Novel strategies to synthesize small genes

In order to develop an efficient, cost-effective and low error rate strategy to produce synthetic genes with reduced size, two different strategies to construct optimized DNA sequences were initially explored: (A) Polymerase chain assembly using DNA template (PCA-DT) and (B) Polymerase chain assembly DNA template-free (PCA-DTF) (Fig. 1a and b, respectively). PCA-DT was developed to decrease the time involved in traditional gene synthesis methods since this method combines both synthesis and cloning in a single reaction. In step 1, two long (A1) or a pool of small oligonucleotides containing the gene sequence (A2 and A3) were mixed with the cloning vector and then assembly proceeds using a DNA polymerase in a typical cyclic temperature reaction (Fig. 1a) (Table 1). In step 2, the product of the PCR amplification combining both the newly synthesized gene and the vector were used to transform *E. coli* cells. PCA-DTF strategy is based on previously reported methods used to produce synthetic genes in a single PCR reaction [4, 9, 11]. All oligonucleotides (inner and outer) were pooled together and assembled in a single polymerase chain reaction. The outer primers were used in a higher concentration than inner primers to ensure the construction of full-length sequence of synthetic gene. Different approaches for oligonucleotide design were tested (Fig. 1, B1, B2 and B3). The PCA-DTF method requires a subsequent ligation-free cloning step to insert the synthetic gene into the cloning vector.

**Table 1:** Gene assembly strategies using for production of the synthetic gene A

Gene assembly method	DNA template	Number of primers	Primer size (nt)	Cloning method
PCA-DT_A1	yes	2	135	Homologous recombination
PCA-DT_A2	yes	13	27–40	Homologous recombination
PCA-DT_A3	yes	12	35–40	Homologous recombination
PCA-DTF_B1	no	14	35–40	Gateway system
PCA-DTF_B2	no	12	30–40	Gateway system
PCA-DTF_B3	no	8	35–60	Gateway system

(A)  PCA-DT
  Three different strategies based on PCA-DT method were used to synthesize gene A (290 nt). Two, fourteen and twelve oligonucleotides were designed to synthesize full-length gene A following strategies A1, A2 and A3, respectively. For A1 strategy, the gene sequence was dissected in two long oligonucleotides of 135 nt, including a 20 nt overlap with the cloning vector. To produce the synthetic gene using A2 strategy, fourteen oligonucleotides of 20–40 nt with a 20 bp overlap region between forward and reverse primers and no gaps between adjacent oligonucleotides were designed. The length of twelve oligonucleotides used in A3 was 35–40 nt and the overlapping region between successive oligonucleotides was 20 bases. All outer primers contained an additional 15-bp homologous sequence to facilitate the homologous recombination reaction associated with *E. coli* transformation. Plasmid pNZY28 was used as a cloning vector and was linearized with *EcoR* V restriction enzyme during 2 h at 37 °C in a heating block. A typical digestion was performed in 100 μL containing 2 μg of plasmid DNA and 50 units of *EcoR* V restriction enzyme. Linear plasmid DNA was purified using silica-based columns, eluted in 50 μL elution buffer and diluted to a final concentration of 20 ng/ μL. The synthesis of gene A using the strategy A1 was initiated with the addition of the two outer primers at a final concentration of 200 nM to 20 ng of digested pNZY28 vector. For strategies A2 and A3, outer and inner primers were used at a final 800 nM and 30 nM concentration, respectively. The PCR reaction was carried out with 200 μM dNTPs and 2.5 units of Pfu Turbo DNA polymerase (Agilent Technologies). The PCR conditions were 30 cycles at 95 °C for 50 s, 50 °C for 50 s and 72 °C for 3 min. The final cycle was followed by an additional 10 min at 72 °C to ensure complete extension of the 3,099 bp gene product (pUC18: 2,886 bp + gene A: 213 bp). PCR amplification products were analysed by agarose gel electrophoresis.
(B)  PCA-FDT
  To synthesize gene A based on strategies B1 and B2 of PCA-DTF, fourteen and twelve oligonucleotides with 40 nt in length and 15 or 20 nt end-overlaps between consecutive oligonucleotides, respectively, were designed (Fig. 1, B1 and B2). Strategy B3 used larger oligonucleotides with 60 nt including 20 nt overlaps (Fig. 1, B3). Outer primers include an additional ligation independent sequence (27 nt on the forward primer and 32 nt on the reverse primer), in order to allow ligation-independent cloning and a 18 bp encoding the Tobacco Etch Virus (TEV) protease. Oligonucleotide assembly was performed as described above using Pfu Turbo DNA polymerase. Assembly reaction was subjected to one cycle of initial denaturation at 95 °C for 5 min, followed by 26 cycles of denaturation at 95 °C for 30 s; annealing at 55 °C for 30 s; and extension at 72 °C for 30 s. PCR amplification products were column purified, as described above, and cloned into pDONR201 vector using Gateway technology (see below).

### Optimization of PCR conditions for successful gene synthesis protocol

Efficacy and accuracy of DNA polymerases and the quality and concentration of primers are two critical parameters known to influence gene synthesis. In addition, annealing temperatures and times used for denaturation, annealing and extension during PCR may affect nucleic acid yields. To optimize these parameters, two genes (B and C) were synthesised using gene synthesis strategy B3. Six and eight oligonucleotides with 58–60 nt and a 20 bp gap between primers were used to synthesise genes B and C, respectively. The sequences of overlapping oligonucleotides used to produce genes B and C are presented in Additional file 3: Table S3. The effect of each PCR parameter was singly tested and the remaining components of PCR reaction were fixed in the standard conditions described above. Four DNA polymerases were selected for these studies: KOD Hot Start DNA polymerase (EMD-Millipore), Q5® Hot Star High Fidelity DNA polymerase (New England Biolabs), Pfu Turbo DNA polymerase (Agilent Technologies) and Taq DNA polymerase (Sigma-Aldrich). PCR was developed in a 26-cycle reaction. Denaturation was performed at 95 °C for 30 s for Pfu Turbo and Taq, 95 °C for 16 s for KOD Hot Start DNA polymerase and 98 °C for 10 s for Q5® Hot Star High Fidelity DNA polymerase. Annealing occurred at 60 °C for 10 s for KOD Hot Start DNA polymerase and 60 °C for 30 s for Q5® Hot Star High Fidelity DNA polymerase, Pfu Turbo and Taq. Finally, extension was performed at 70 °C for 3 s for KOD Hot Start DNA polymerase, 72 °C for 30 s for Pfu Turbo and Taq, and 72 °C for 15 s for Q5® Hot Star High Fidelity DNA polymerase. Different overlapping oligonucleotide concentrations were tested. Gene assembly was performed using inner oligonucleotides at a final concentration of 10, 20 and 30 nM. Outer primers were used at final concentrations of 200, 600, 800 and 1000 nM. In addition, the final concentration of dNTPs was set to vary between 0.1 and 0.5 mM. Finally, different PCR profiles were tested. Thus, PCRs were performed at five different annealing temperatures (from 50 °C to 62 °C). Furthermore, a total of 24 PCR programs, which included different times of denaturation, annealing and extension in a total of 22, 24 and 26 cycles, were tested. Final configuration of each PCR program used in these studies is presented in Additional file 4: Table S4.

### Cloning and sequencing

After PCR assembly, resulting nucleic acids were purified, inserted into a suitable vector and the integrity of each gene was confirmed by DNA sanger sequencing. Synthesized genes produced by strategy A were ligated into pNZY28 cloning plasmid using the homologous recombination machinery present in *E. coli* cells. Gene products from strategies B1, B2 and B3 which contained attb1 and attb2 sequences at its 5’ and 3’- ends, respectively, were cloned into pDONR201 vector (ThermoFisher Scientific) using the Gateway cloning system (ThermoFisher Scientific). Cloning reaction mixtures were used to transform *E. coli* DH5α competent cells. For each transformation, one bacterial colony was inoculated and grown in liquid LB medium supplemented with 100 μg/mL of ampicillin. Plasmids were purified and recombinant integrity of inserted nucleic acids confirmed by sequencing.

### Construction of a novel gene synthesis platform for the large scale production of small synthetic genes

An integrated gene synthesis platform was developed for the efficient production of small synthetic genes. This platform combines automation, simplicity and robustness, while decreasing the error rate associated with conventional gene synthesis methods. Initial experiments described above defined the most appropriate PCR assembly protocol. Subsequent experiments evaluated the efficacy of the protocol when applied for the simultaneously synthesis of 96 genes encoding venom peptides. Ninety six genes were designed by back-translating corresponding peptide sequences and by optimizing codon usage for high levels of expression in *E. coli*. Codons were selected randomly using a Monte Carlo approach according to *E. coli* codon usage of highly expressed genes. Genes were designed to have a GC content between 40 and 60% and a codon adaptation index (CAI) value higher than 0.8. The sequences of the 96 optimized genes are presented in Additional file 5: Table S5. The pool of primers required to synthesize 96 synthetic genes encoding venom peptides were designed to have 50–60 nt in length, an overlap region of 20 nt between forward and reverse primers with a gap sequence of 20 nt, and included an additional 16-bp conserved sequences at 5’- terminus of both forward and reverse outer primers to allow ligation-independent cloning. The 96 genes were produced from 96 mixes of six oligonucleotides with 50–60 nt in length and 20 nt overlaps. Oligonucleotides were synthesised by Integrated DNA Technologies at the smallest scale (primer solutions at 5 μM) with desalting purification. The two outer primers were used at a final concentration of 800 nM while the inner primers were pooled together in an equimolar mixture to achieve the final concentration of 20 nM. Primer dilutions and PCR assembly was carried out in a 96-well plate format using a Tecan workstation (Switzerland). KOD Hot Start DNA polymerase (EMD Millipore) was used for PCR assembly using optimized conditions to minimize primer-dimer formation and nonspecific amplifications. PCR reactions were performed in a 50 μL total volume and consisted of 0.2 mM dNTPs, 1.5 mM MgCl_2_, 1× reaction buffer and 1 unit of KOD Hot Start DNA polymerase. PCR assembly reactions were carried out in a 96-well PCR plate format. The cycling parameters were as follows: 1 cycle of 95 °C for 2 min; 26 cycles of 95 °C for 20 s, 60 °C for 8 s, and 70 °C for 3 s. After PCR assembly, assembled PCR products were visualized by agarose gel electrophoresis and purified through silica-base chromatography in a Tecan liquid handler (Switzerland). Purified PCR products were cloned into pHTP1 expression vector (NZYTech, Ltd) using the NZYEasy cloning kit (NZYTech, Ltd) that follows a LIC technology. Gene assembly products were mixed with 120 ng of linearized vector, using a molar ratio of 1:5 (vector:insert). Cloning reactions were performed in 10 μL volume in a 96-well PCR plate and preceded for 1 h at 37 °C on a heating block. The mixtures were then incubated at 80 °C for 10 min followed by 10 min at 30 °C. Recombinant plasmids were transformed using a high-throughput method into *E. coli* DH5α competent cells and spread on LB agar plates supplemented with 50 μg/mL of kanamycin. After overnight incubation at 37 °C, only one colony per transformation was picked and grown in 5 mL of LB kanamycin medium in 24-deep-well plates sealed with gas-permeable adhesive seals. Cultures were incubated at 37 °C for ~16 h and cells were then harvested at 1,500 *× g* for 15 min. Plasmids were purified from bacterial pellets in a Tecan workstation (Switzerland), and subsequently the DNA sequence of each gene was verified by Sanger sequencing. In case the DNA sequence did not correspond with the designed gene, a second and eventually third colony was picked for sequencing analysis.

## Results and discussion

### Synthesis and assembly of the 213 nt gene encoding alpha-elapitoxin-Nk2a toxin using PCA-DT and PCA-DTF methods

**Fig. 2:**
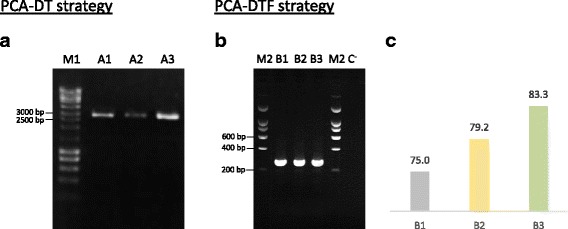
The efficacy of PCA-DT and PCA-DTF methodologies to generate small nucleic acids. Panel **a**: synthesis of gene A was performed using a vector DNA template (A1, A2 and A3) and the PCR products correspond to a 3,099 bp DNA fragment, which combines gene A sequence plus pNZY28 vector sequence. **b**: gene A was also assembled using different pools of overlapping oligonucleotides (B1, B2 and B3); lane C^−^ corresponds to a negative control reaction performed at the same conditions, without addition of primers. **c**: Effect of primer design on percentage of clones without errors. M1: NZYLadder III, M2: NZYLadder I

The gene encoding the toxic peptide *alpha-elapitoxin-Nk2a* was designed to maximize expression in *E. coli*. Initial experiments aimed at identifying the most appropriate strategy for the synthesis of small genes and attempted to reduce the number of steps involved in traditional gene synthesis approaches. Thus, the efficiency of PCA-DT and PCA-DTF PCR-based methods was tested for the synthesis of gene A, which has a size of 290 nt (Fig. 1). To ensure simplicity and speed in the synthetic gene process, PCA-DT does not involve an additional cloning step. Using method A1 (Fig. 1), gene A was synthesised using pNZY28 vector as DNA template and employing two long oligonucleotides (135 nt) to amplify the full-length plasmid sequence containing half of the gene sequence in each 5’ and 3’ ends. Long oligonucleotides are more prone to incorporate errors. Thus, in alternative, gene A was also amplified using a set of 14 and 12 overlapping oligonucleotides (Fig. 1, strategies A2 and A3, respectively), which contain a 20 nt sequence that hybridises with the cloning plasmid. In contrast, PCA-DTF methods use a template-less approach to assemble the artificial gene. The six methods described in Fig. 1 were employed to synthesize gene A. The data, presented in Fig. 2, revealed that all strategies effectively generated the toxic gene. However, yield of the assembled target nucleic acid was higher using strategies B when compared with the quantity of PCR product obtained using the PCA-DT. Although strategy A does not require an additional step to insert the synthetic gene into the cloning plasmid, globally the process is more tedious due to the long periods required for the amplification of large nucleic acids, as PCR involves both the toxic gene and the vector (~3 Kb, pNZY28 vector plus target gene). In addition, cloning efficiency, evaluated through colony-PCR, revealed an approximately 95% cloning efficacy of the Gateway system versus 80% when using the self-ligation process of strategy A. These results suggest that strategies including a DNA template and involving plasmid re-circularization are probably not the best option for the synthesis of small genes as they are more tedious while leading to lower cloning efficiencies. These two issues are particularly important when protocols require automation.

In order to verify if the length of the oligonucleotides influences the appearance of errors in synthetic genes, we selected 24 clones synthesized by strategies B1, B2 and B3 for DNA sequence verification using Sanger sequencing. Analysis of the 72 sequences revealed that approximately 80% of the clones for each one of the three strategies presented the correct DNA sequence (Fig. 2c). Thus, increasing primer size from 40 to 60 nt has no impact in the number of errors in the resulting synthetic DNA. This primer size was also used by Stemmer and colleagues to successfully synthesise two long DNA fragments [4], although the error rate associated with the method was not reported. Therefore, taken together, the data suggest that the PCA-DTF gene assembly method that uses a set of 60 nt overlapping oligonucleotides with 20 nt gaps, is the most convenient strategy for the synthesis of small genes as it provides high gene synthesis yields and efficacy with increased cloning efficiencies.

### Performance of various thermostable DNA polymerases for gene synthesis

**Fig. 3:**
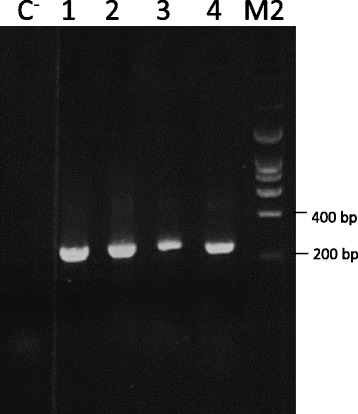
Performance of four thermostable DNA polymerases for the synthesis of gene B using PCA-DTF. Lane 1: negative control reaction performed at the same conditions, without addition of primers; lane 2: KOD Hot Start DNA polymerase; lane 3: Q5 Hot Start High Fidelity DNA polymerase; lane 4: Pfu Turbo DNA polymerase and lane 5: Taq DNA polymerase. M2: NZYladder I

*Taq, Kod* and *Pfu* polymerases have been commonly used for the production of synthetic genes following PCR-based methods [4, 7, 13]. However, *Taq* polymerase is known to be error-prone [14]. The use of *Kod* and *Pfu* polymerases allow higher accuracy in gene synthesis although the elongation rate of *Pfu* polymerases is lower [15]. Here, the efficacy of four different DNA polymerases (KOD Hot Start DNA polymerase, Q5® Hot Start HF, Pfu Turbo DNA polymerase, and Taq DNA polymerase) for the production of gene B (260 bp), using B3 strategy PCA-DTF (described above) was analysed. The data, presented in Fig. 3, revealed that the four polymerases effectively assembled the 260 bp gene. However, KOD Hot Star DNA polymerase seems to express a higher performance when compared with the other enzymes. These data suggest that efficacy of *Kod* polymerases is higher than *Taq* and *Pfu* enzymes for assembling small genes. After cleaning the PCR products, genes were cloned into pDONR201 vector and 24 clones assembled by each one of the four DNA polymerase were sequenced. The data, presented in Table 2, revealed that appearance of mutations is more frequent for *Taq*, followed by Q5® Hot Start HF and *Pfu* Turbo DNA polymerase. In contrast, only five out of the 22 recombinant plasmids containing the synthetic gene B assembled by KOD Hot Star DNA polymerase presented errors, which reflects one mutation per 1.15 kb. Deletions and substitutions were the most frequent errors identified in the 96 variants of gene B sequenced. As expected, the data suggested that KOD Hot Start DNA polymerase is more accurate than the other three DNA polymerases due to a higher fidelity. These results are in line with previous studies [8, 9, 16], which have revealed that usage of high fidelity DNA polymerases decreases the number of errors introduced in synthetic genes during PCR amplification. Moreover, the PCA-DTF procedure appears to be very efficient with KOD Hot Start DNA polymerase due to the rapid elongation rates presented by this DNA polymerase; completion of the gene synthesis protocol is achieved in less than 40 min.

**Table 2:** Gene synthesis error rates when were used different DNA polymerases

DNA polymerase	Hot Start activity	Number of clones sequenced	Bases sequenced	Number of bases deleted, inserted or substituted	Error rate (error/kb)	Number of deletions	Number of insertions	Number of substitutions
KOD HS DNA pol.	yes	24	5720	5	0.87	2	1	2
Q5® HS HF DNA pol.	yes	24	6240	10	1.6	6	1	3
NZYProof DNA pol.	no	24	6240	10	1.6	5	2	3
NZYTaq DNA pol.	no	24	5980	13	2.2	2	3	7
Total		96	24,180	38		15	7	15

### Oligonucleotide concentration influences the efficacy of gene synthesis

**Fig. 4:**
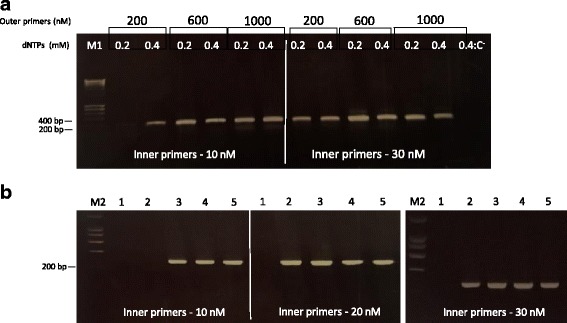
Influence of oligonucleotide concentration in the efficacy of the assembly reaction. **a**: PCR assembly was performed with 200, 600 and 1000 nM of outer oligonucleotides using 0.2 or 0.4 mM dNTPs and 10 nM or 30 nM of inner primers. A control reaction containing no primers was performed under same conditions (outer primers concentration:1000 nM and 0.4 mM of dNTPs). **b**: Outer primers concentration was fixed to 800 nM and inner primer concentration varied between 10 to 30 nM. Lanes 1–5 correspond to: 0.1, 0.2, 0.3, 0.4 and 0.5 mM dNTPs. M1: NZYLadder III; M2: NZYLadder I

Since PCA-DTF is suggested to be the best method to produce small synthetic genes and KOD Hot Star DNA polymerase is the most effective enzyme to apply in these protocols, we analysed the influence of oligonucleotide concentration on gene assembly efficiency. Thus, three different concentrations of inner oligonucleotides were combined with different concentrations of outer primers in a PCR-assembly reaction set to synthesize gene B. Initially, concentrations of inner oligonucleotides were of 10 nM and 30 nM, and outer oligonucleotides were tested at 200, 600 and 1000 nM. After the assembly of gene B the resulting nucleic acids were analysed by agarose gel electrophoresis. The data, presented in Fig. 4a, suggest that gene synthesis is most effective with 30 nM of inner primers. In addition, the best concentrations of outer primers were of 600 and 1000 nM. In order to define more precisely the best concentrations of primers, outer primer concentration was fixed at 800 nM and concentrations of inner primers varied from 10 to 30 nM. Data suggest that at 800 nM of outer primers, the optimal concentration of inner oligonucleotides is 20 nM (Fig. 4b). The assembly reaction was also performed under different concentrations of dNTPs. Interestingly, the results suggest that concentrations of dNTPs below 0.2 mM are not appropriate for gene synthesis. Thus, for a robust and successful PCA-DTF procedure a concentration of 0.3–0.4 mM of dNTPs seems to be the most effective (Fig. 4).

### Effect of cycling temperatures on the efficiency of gene synthesis

**Fig. 5:**
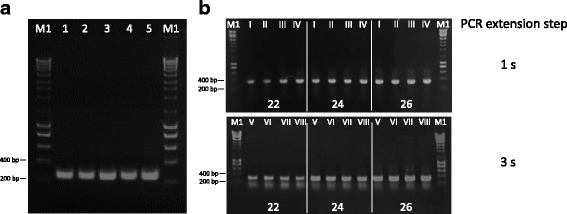
Effect of cycling temperatures on the efficiency of gene synthesis. Panel **a**: different annealing temperatures were studied (1: 50 °C; 2: 52.3 °C; 3: 54.6 °C; 4: 59.6 °C and 5: 62 °C). Panel **b**: 22, 24 and 26 cycles combined with different times of denaturation, annealing and extension were used to synthesise gene C (I: 95 °C for 20 s, 60 °C for 10 s, 70 °C for 1 s; II: 95 °C for 16 s, 60 °C for 10 s, 70 °C for 1 s; III: 95 °C for 20 s, 60 °C for 8 s, 70 °C for 1 s; IV: 95 °C for 16 s, 60 °C for 8 s, 70 °C for 1 s; V: 95 °C for 20 s, 60 °C for 10 s, 70 °C for 3 s; VI: 95 °C for 16 s, 60 °C for 10 s, 70 °C for 3 s; VII: 95 °C for 20 s, 60 °C for 8 s, 70 °C for 3 s; VIII: 95 °C for 16 s, 60 °C for 8 s, 70 °C for 3 s). The extension times used for each number of cycles were 1 and 3 s

In the previous assembly reactions following PCA-DTF, annealing temperature was set to 60 °C. Several studies [3] have suggested that factors such as melting temperature (T_m_) and GC content affect optimal assembly. Thus, the efficiency of synthesis of gene B was tested using a gradient of annealing temperatures (50 °C, 52.3 °C, 54.6 °C, 59.6 °C and 62 °C) applying the optimal PCR conditions described in the previous section. The data, presented in Fig. 5, suggest that oligonucleotide assembly occurs at temperatures ranging from 50 to 62 °C, although yields of nucleic acid seems to increase at higher annealing temperatures. In addition, the effect of the number of PCR cycles in the efficiency of gene synthesis was tested by synthesizing gene C using 22, 24 and 26 thermal cycles. The data revealed that, as expected, the quantity of the amplified product increases with the number of thermal cycles employed (Fig. 5). Likewise, when denaturation last 20 s, an extension of 3 s produces higher DNA yields than a 1 s extension. In addition, annealing of overlapping oligonucleotides during 8 s is more favourable than for 10 s. Therefore, the data revealed that 26 thermal cycles of denaturation (20 s), annealing (8 s) and extension (3 s) step are optimal for PCR assembly following the PCA-DTF method.

### Effect of oligonucleotide source in the efficacy of gene synthesis

**Fig. 6:**
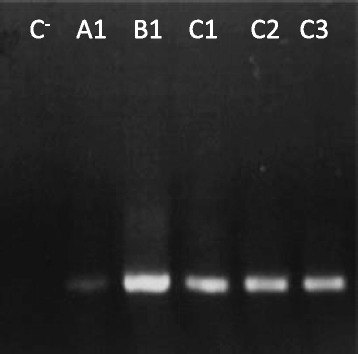
Assembly of gene C using oligonucleotides obtained from three different suppliers (A, B and C) and three purification methods. Numbers correspond to desalted (1), reverse-phase cartridge (2) and HPLC (3) primers purification methods. Lane 1 corresponds to a negative control reaction performed without addition of oligonucleotides

It is well known that efficacy of gene synthesis directly depends on the quality of the synthetic oligonucleotides used for DNA assembly [17]. Current chemical synthesis methods usually produce oligonucleotides that are prematurely terminated or comprise internal insertions or deletions [17]. To determine how the oligonucleotide source modulates the production of error-free DNA fragments, gene C was synthesised using desalted, reverse-phase cartridge and reverse-phase HPLC purified primers obtained from three different suppliers. Gene C was assembled using PCR conditions defined above and five different oligonucleotide sources were analysed. The results, presented in Fig. 6, show that oligonucleotides from supplier B displayed the best performance. DNA plasmids of 16 recombinant clones for each condition were analysed by Sanger sequencing. The highest percentage of clones without errors was identified in genes synthesized with primers for supplier B which were not subjected to any purification (Table 3). PCR products assembled using reverse-phase cartridge and HPLC oligonucleotides have a lower percentage of clones without errors (B2 - 50% and B3 - 56%, respectively) when compared with exclusively desalted oligonucleotides. Thus, it is noteworthy observing that oligonucleotide purification does not solve the percentage of mutation observed in artificial genes. The most frequent mutation identified in the 80 recombinant clones was a single base deletion (44%, see Table 3 and Additional file 6: Table S6). These results suggest that truncated versions of the oligonucleotides (*n-1*) are difficult to remove by accessory purification methods as the desalted oligonucleotides from supplier B contain a lower frequency of deletions. In contrast, previous studies have shown that PAGE oligonucleotide purification is recommended for the successful production of synthetic genes by PCR assembly [8, 13, 16, 18]. However, the error rates reported in these studies is identical (~1 error per Kb of synthetic DNA) to our gene synthesis method revealing that oligonucleotide purification is not crucial for the accurate production of DNA fragments. In our high-throughput study, the purification of oligonucleotides can be a disadvantage since the production cost would significantly increase.

**Table 3:** Oligonucleotides purification and source used to assembly the gene C

Oligonucleotide source	Supplier	Purification	Number of clones sequenced	Clones without errors	Number of bases deleted, inserted or substituted	Number of deletions	Number of insertions	Number of substitutions
A1	A	desalted	16	68%	6	4	1	1
B1	B	desalted	16	80%	3	2	0	1
C1	C	desalted	16	71%	7	3	2	2
C2	C	cartridge	16	50%	14	4	5	5
C3	C	HPLC	16	56%	11	5	4	2
Total			80		41	18 (18/41 = 44%)	12 (12/41 = 29%)	11 (11/41 = 27%)

### Large scale synthesis of genes encoding venom peptides using an automated platform

**Fig. 7:**
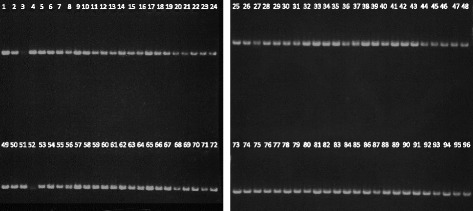
Agarose gel electrophoresis of 96 nucleic acids encoding venom peptides assembled simultaneously using a large scale gene synthesis platform. 98% of gene products presented high yield and correct size. 2 out the 96 genes (3 and 52) showed PCR fragments with low yield. However, the 96 PCR products were used for subsequent cloning reaction and resulting plasmids were employed to transform *E. coli* cells. Resulting recombinant plasmids were purified and the integrity of DNA sequences was verified by Sanger sequencing

Previous optimized protocols were used to develop a platform for the simultaneous synthesis of small genes. Thus, the primary sequence of 96 venom peptides was used to design 96 genes that contained an average GC content of 49% and an average CAI of 0.86 (Table 4). To assemble the 96 genes, 576 (6 primers × 96 genes) oligonucleotides with a maximum of 60 nt were designed with an overlap region of 20 bp and a gap of 20 bp. In average, the genes had 240 bp in length and oligonucleotides were acquired without additional purification. Each gene was PCR assembled using the KOD Hot Start DNA polymerase. Outer primers were used at 800 nM (forward and reverse) while inner primers at a final concentration of 20 nM. PCR assembly was performed in 26 cycles of 95 °C for 20 s, 60 °C for 8 s and 3 s at 70 °C. The 96 genes were assembled simultaneously in a 96-well PCR plate and resulting nucleic acids analysed through agarose gel electrophoresis. The data, presented in Fig. 7, revealed that 94 out of the 96 genes were effectively assembled representing a 98% success rate of the gene synthesis protocol when applied to a large scale. After purification of the 96 generated PCR products, individual genes were sub-cloned into pHTP1 expression vector using a LIC method. The robustness and effectiveness of the pipeline was demonstrated when recombinant plasmids were sequenced to verify gene integrity. The initial screen of one clone per gene revealed that 77 genes (80.2%) were correct (Table 5). For 17 genes (17.7%) two clones were screened to identify an error-free DNA fragment. Finally, for 2 genes (2.1%) it was necessary to pick a third clone to obtain a correct DNA sequence. Thus, even for the two genes that apparently were amplified at a lower concentration it was possible to obtain a correct clone. In total, 26 mutations were identified in incorrect genes, leading to an overall error rate of 1 mutation per 0.9 kb. The majority of the identified mutations were deletions (77%), as it is expected from the incorporation of prematurely terminated oligonucleotide [19]. The remaining mutations were single-base substitutions (19%) and insertions (4%).

**Table 4:** Properties of 96 optimized genes that were synthesised using the HTP gene synthesis platform

	Length (nt)	GC content (%)	Codon adaptation index (CAI)
Mean (±SD)	240 ± 9	48.9 ± 3.4	0.86 ± 0.03
Maximum	254	57	0.97
Minimum	215	40	0.80

**Table 5:** Properties of primers used in gene synthesis of 96 genes encoding venom peptides. Error rate determined for the integrated HTP gene synthesis platform developed in this study

Number of genes	Number of primers in the PCR assembly	Primers lengths (nt)	Primers overlaps/gaps (nt)	Bases sequenced	Number of bases deleted, inserted or substituted	Error rate (error/kb)	Number of deletions	Number of insertions	Number of substitutions	Genes with 1 clone sequenced	Genes with 2 clones sequenced	Genes with 3 clones sequenced
96	6	57–60	20	23,058	26	1.13	20	1	5	77	17	2

There are some major differences between the approach and resulting efficacy of the method reported here when compared with previously described protocols (Table 6). Firstly, some of the reported gene synthesis methods involve usage of oligonucleotides subjected to subsequent downstream purifications (see Table 6). The protocol described here uses desalted oligonucleotides (no purification) to accurately produce different DNA fragments. Since price of non-purified oligonucleotides is reduced, the cost associated with the method described here is lower, which represents a strong advantage for both low and high-throughput protocols. Secondly, this method uses 60-bp oligonucleotides while some reported methods use oligonucleotides with a length below 60 bases. Thus, the number of oligonucleotides required for each assembly reaction using the protocol described here is significantly reduced while maintaining the success rate of gene synthesis. Although large primers can incorporate more errors, data reported here show that the increase of oligonucleotide size from 40 to 60 bp had no impact in the percentage of correct synthesised DNA sequences. Thus, for the protocol reported here, the use of 60-bp oligonucleotides is believed to provide the best balance between error rate and production cost. Finally, the error rate observed in the gene synthesis method reported here is lower or identical to previously reported methods (Table 6), revealing that this platform is efficient and robust to synthesise multiple genes in simultaneous.

**Table 6:** Comparison of different methods used to produce synthetic DNA fragments

Year	Method	Number of steps for gene synthesis	Oligo length	Oligo overlap	Oligo gap	Oligo purification	DNA polymerase	Accuracy or error rate per Kb	Reference
2016	This study	Single step: assembly PCR	60	20	20	No purification	*Kod* Hot Start	1.13	–
1995	Stemmer method	Single step: assembly PCR	40	20	NM	No purification	*Taq*	NM	[4]
2004	PTDS	Two steps: Assembly PCR & Overlap extension PCR	60	20	NM	PAGE	*Pfu* or *pyrobest*	1.26	[13]
2004	Two-step method	Two steps: Dual asymmetric PCR & Overlap extension PCR	50	10	NM	No purification	*Pfu*	3/4 clones contain 1–3 single base deletions	[7]
2006	PAS	Two steps: Assembly PCR & Overlap extension PCR	60	21	NM	PAGE	*Pfu*	~1	[18]
2006	SGS	Single step: assembly PCR	40	18–20	No	No purification	*Kod*	2.7	[9]
2010	IPS	Two steps: Assembly PCR & Overlap extension PCR	60 & 30	15	NM	PAGE	*Pfu*	1	[8]
2012	AOE	Two steps: Assembly PCR & Overlap extension PCR	30–50	NM	NM	PAGE	*Pfu*	<1	[19]
2015	RapGene	Single step: assembly PCR	57–60	20	NM	No purification	NM	1.67	[20]

NM-not mentioned

## Conclusions

The ability to *de novo* synthesize DNA sequences is rapidly emerging to improve the speed, accuracy and simplicity of recombinant DNA technology. Here, we have optimized a novel gene synthesis large scale platform for the efficient production of small genes (<0,5 kb). The genes were directly cloned into an *E. coli* expression vector using a completely automated protocol. This gene synthesis approach presents high efficiencies of PCR assembly and cloning while revealing low error rates. The error rate of the large scale method described here is of 1.1 mutations per kb. Low error rates avoid additional steps for the removal of errors from synthesized genes, such as those involving the use of proteins that recognize mismatches within DNA sequences to remove DNA mutations. The identification of 100% correct genes was performed by screening a maximum of 3 colonies. Thus, the labour required for the selection and validation of recombinant clones is reduced. The use of overlapping oligonucleotides combined with *Kod* DNA polymerase provides a powerful alternative to conventional synthesis protocols. The length of all oligonucleotides is below 60 nt, with 20-bp overlap regions and gaps of 20 nt. This represents a decrease in the number of oligonucleotides for a given gene saving costs. The PCR-based gene synthesis method described here is an optimization of the simplified gene synthesis method (SGS) [9]. However, among other details, average primer lengths in the protocol described in this study is larger than used in SGS methods. In conclusion, the gene synthesis approach described here is a simple, accurate and robust system that can be used to construct at low cost and in short periods of time large numbers of *de novo* DNA molecules for a variety of applications.

## Additional files

Additional file 1: Table S1. Sequences properties of genes A, B and C used to optimize the PCR conditions. (XLSX 10 kb)
Additional file 2: Table S2. Primer sequences used to assembly of gene A using different approaches for gene synthesis. (XLSX 12 kb)
Additional file 3: Table S3. Primer sequences used to assembly of genes B and C. (XLSX 9 kb)
Additional file 4: Table S4. PCR programs used to optimize the PCR profile of a PCR-assembly reaction. (XLSX 9 kb)
Additional file 5: Table S5. Sequence properties of 96 genes synthesised using the HTP gene synthesis platform. (XLSX 23 kb)
Additional file 6: Table S6. Mutation types identified in the genes produced. (XLSX 15 kb)

## Abbreviations

CAI: Codon Adaptation Index
DA-PCR: Dual asymmetrical PCR
GC: Guanine-cytosine
IPS: Improved PCR synthesis
Kb: Kilobase
LIC: Ligation-Independent Cloning protocol
Nt: Nucleotides
OE-PCR: Overlap-extension PCR
PAS: PCR-based accurate synthesis
PCA: Polymerase chain assembly
PCA-DT: Polymerase chain assembly using DNA template
PCA-DTF: Polymerase chain assembly DNA template-free
PTDS: PCR-based two-step DNA synthesis
SGS: Simplified gene synthesis
TBIO: Thermodynamically balanced inside-out technology
TEV: Tobacco Etch Virus protease.
